# 3-weekly or weekly cisplatin concurrently with radiotherapy for patients with squamous cell carcinoma of the head and neck – a multicentre, retrospective analysis

**DOI:** 10.1186/s13014-019-1235-y

**Published:** 2019-02-11

**Authors:** Seth Helfenstein, Oliver Riesterer, Urs R. Meier, Alexandros Papachristofilou, Benjamin Kasenda, Miklos Pless, Sacha I. Rothschild

**Affiliations:** 1 Department Internal Medicine, University Hospital Basel, Medical Oncology, Petersgraben 4, 4031 Basel, Switzerland; 2 Clinic for Radiation Oncology, University Hospital and University of Zürich, Rämistrasse 100, 8091 Zürich, Switzerland; 3 Cantonal Hospital Winterthur, Clinic for Radiation Oncology, Brauerstrasse 15, 8400 Winterthur, Switzerland; 4 Clinic for Radiotherapy and Radio-Oncology, University Hospital Basel, Petersgraben 4, 4031 Basel, Switzerland; 5 Medical Oncology, Cantonal Hospital Winterthur, Brauerstrasse 15, 8400 Winterthur, Switzerland

**Keywords:** Head and neck squamous carcinoma, Chemo-radiotherapy, Treatment, Cisplatin, Dose

## Abstract

**Background:**

Concurrent chemoradiotherapy with cisplatin is standard for patients (pts) with loco-regionally advanced squamous cell carcinoma of the head and neck (LA-SCCHN) and for patients with resected SCCHN with high-risk features. The standard regimen includes 3-weekly cisplatin, but weekly regimens are often used to lower toxicity. Reaching a cumulative dose of ≥200 mg/m^2^ cisplatin was shown being associated with improved outcome. We herein investigated cumulative dose reached and toxicities between the 3-weekly and weekly cisplatin regimens with concurrent radiotherapy.

**Methods:**

Multicentre, retrospective analysis of all patients undergoing combined RCT with cisplatin treated at 3 centres in Switzerland between 06/2008 and 12/2015.

**Results:**

Three hundred fourteen pts. were included (3-weekly, *N* = 127; weekly, *N* = 187). Median cumulative cisplatin dose was 200 mg/m^2^ (IQR 150–300) for pts. treated with a 3-weekly schedule and 160 mg/m^2^ (120–240) for the weekly schedule, consequently more pts. treated with a 3-weekly schedule reached a cumulative dose ≥200 mg/m^2^ (75.6% vs. 47.1%, *p* < 0.001). This association was also observed in multivariable analysis adjusted for age and sex (OR 3.46, 95% confidence interval [CI], 2.1–5.7). The 3-weekly regimen led to a higher rate of acute renal toxicity (33.1% vs. 20.9%, *p* = 0.022). In the landmark analysis, we could not confirm that a cisplatin dose ≥200 mg/m^2^ is associated with better survival (HR 1.3, 95% CI 0.8–1.9).

**Conclusions:**

Significantly more patients receive a cumulative cisplatin dose of ≥200 mg/m^2^, when treated with a 3-weekly schedule compared to weekly dosing. The previously reported association between a cumulative cisplatin dose ≥200 mg/m^2^ and improved outcome could not be shown in our study.

## Background

Prognosis of patients with squamous cell carcinoma of the head and neck (SCCHN) has improved in the last decades [1–4]. However, most SCCHN patients are diagnosed with loco-regionally advanced (LA-SCCHN) stage disease. In this setting, multimodal therapy including combined radiochemotherapy (RCT) either as adjuvant therapy after tumor resection or as definitive curatively intended treatment approach is recommended. Several Phase III randomized trials confirmed improved loco-regional control and longer survival for high-risk patients with combined versus sequential RCT with cisplatin in the definitive [5–7] and postoperative setting [8–10]. For non-surgical cases the meta-analysis by Pignon et al. including 93 trials and more than 17′000 patients demonstrated an overall survival (OS) benefit from the addition of chemotherapy of 6.5% at five years compared to radiotherapy (RT) alone [11]. In a second meta-analysis, these results were confirmed and it was demonstrated that concurrent (but not sequential) RCT improved median OS by one year [12]. The standard treatment regimen consists of three cycles of cisplatin 100 mg/m^2^ given on days 1, 22 and 43 of a 7-week RT course [5, 6, 13]. Due to adverse events (AEs) of this intensive regimen, chemotherapy modifications (dose reductions, delays, and omissions) are required in up to 40% of patients [14, 15]. The main AEs due to cisplatin include renal insufficiency, electrolyte disorders, myelo- and ototoxicity [6, 8, 9, 16]. Suboptimal compliance with cisplatin regimen might negatively impact patient outcome [5, 17, 18]. Therefore, alternative treatment schedules including low-dose cisplatin have been investigated [7, 19–24]. Based on these studies, weekly cisplatin administered at a dose of 30–40 mg/m^2^ is frequently used [7, 19, 21, 22, 24]. Regardless of the treatment regimen, it has been suggested that a cumulative dose of 200 mg/m^2^ needs to be reached for therapeutic benefit [11, 25]. A retrospective analysis revealed inferior outcome with a cumulative cisplatin dose of ≤200 mg/m^2^ in HPV-negative patients. Recently, a randomized phase III non-inferiority trial suggested that a 3-weekly schedule with cisplatin 100 mg/m^2^ results in superior loco-regional control compared to a weekly dose of 30 mg/m^2^ [23]. However, the higher dose regimen was associated with significantly more acute toxicities of grade 3 or higher. Although this is the first randomized trial, the results have to be interpreted with caution as more than 90% of patients were diagnosed with oral cavity tumors and more than 90% of patients were treated with RCT in the postoperative setting. Several other comparisons between different treatment regimens have been made mainly in retrospective [26–32] and small prospective trials [33–35] and showed contradictory findings.

The aim of this multicenter, retrospective analysis was to compare outcome, cumulative cisplatin dose and acute treatment toxicity in patients undergoing RCT for SCCHN receiving 3-weekly or weekly cisplatin concurrently with intensity-modulated radiotherapy (IMRT).

## Methods

### Study population

In this multicentre, retrospective, non-interventional study, we included 314 patients treated at three hospitals in Switzerland (University Hospital Basel (USB), University Hospital Zürich (USZ), Cantonal Hospital Winterthur (KSW)) between June 2008 and December 2015 (USZ: June 2008 and December 2015). All patients underwent a routine staging procedure consisting of physical examination, chest x-ray, computed tomography (CT), magnetic resonance imaging (MRI) or positron emission tomography-computed tomography (PET-CT) scan. Routine laboratory tests consisted of complete blood count and chemistry including renal function.

We included patients (≥ 18 years) if they had squamous cell carcinoma of the head and neck, UICC stage II-IV, either treated with primary definitive RCT or radically resected with high-risk features (R1 resection, extracapsular spread in cervical lymph nodes) and treated with adjuvant RCT. Patients had to have received at least one dose of cisplatin to be included.

### Data collection

Medical charts were reviewed systematically considering demographics and clinical characteristics including age, gender, smoking history, alcohol consumption habits and comorbidities. Stage was assessed based on the American Joint Committee on Cancer (AJCC) TNM version 7 classification. Comprehensive data on radiotherapy and chemotherapy treatment details were collected as well as serial laboratory findings with a focus on hemato- and nephrotoxicity. Clinical assessments were performed at 4, 8 and 12 weeks after the end of treatment, then every three months for the first two years and semi-annually thereafter. Radiographic follow-up (CT, MRI or PET-CT) was performed 8–12 weeks after the end of treatment and then semi-annually. Follow-up data for all patients were obtained until June 2016 or five years after treatment initiation.

### Chemotherapy

Cisplatin was given either at a dose of 100 mg/m^2^ every 3 weeks (day 1, 22, and 43) during RT (University Hospital Basel) to a planned cumulative dose of 300 mg/m^2^ or at a dose of 40 mg/m^2^ (University Hospital Zurich) or 50 mg/m^2^ (Cantonal Hospital Winterthur) weekly for 6 or 7 infusions (days 1, 8, 15, 22, 29, 36, and 43) to a planned cumulative dose of 240–350 mg/m^2^.

### Radiotherapy

All patients received computed tomography (CT) based radiotherapy planning and were treated with IMRT techniques including static field IMRT or volumetric arc radiotherapy (VMAT). Definitive IMRT was given to a total dose of 69 to 72 Gy in 33–36 fractions and adjuvant radiotherapy with 60 to 66 Gy in 30–33 fractions. In general, the first treatment volume included the primary tumor site and elective nodal areas of the bilateral neck. A second treatment volume encompassed the primary tumor site and affected nodes. These volumes were treated either sequentially or simultaneously (simultaneous integrated boost).

### Endpoints

Progression-free survival (PFS) was defined as the time from diagnosis until tumor relapse/progression or death from any cause. Overall survival (OS) was defined as the time from diagnosis until death from any cause. Renal toxicity and ototoxicity as adverse events of special interest (AESI) were assessed during RCT and until the first posttreatment assessment 6 weeks after end of therapy according to the Common Terminology Criteria of Adverse Events (CTCAE) version 4.0 [36]

### Statistical analysis

Our primary objective was to assess the association between the cumulative cisplatin dose achieved and the intended treatment protocol. Secondary objectives included investigation of clinical endpoints such as PFS, OS, total radiation dose and toxicity (ototoxicity and renal toxicity).

We stratified patients according to the treatment applied and used descriptive statistics to compare baseline characteristics and outcomes between these groups. For time-to-event endpoints (PFS and OS), we created Kaplan-Meier plots to visualize the respective effects; the log-rank test was used to test for comparison of survival rates. To investigate the association between the chosen cisplatin regimen (weekly versus 3-weekly; dependent variable) and the chance to reach the cumulative cisplatin dose of ≥200 mg/m^2^ (dependent variable), we used multivariable logistic regression techniques with patient age and gender as potential confounders. We have chosen these two clinical variables to adjust for potential confounding, because we assumed that these baseline factors could influence clinical decision making during treatment and alter the chance to reach the cumulative cisplatin dose > 200 mg/m^2^. To investigate the prognostic impact of the achieved cumulative cisplatin dose (independent variable) on survival, we used a Cox regression model; to account for possible guarantee time bias, a landmark approach was applied only including patients alive 8 weeks after start of treatment. Beside age and gender, we included tumour localization and smoking history to adjust for their potential impact on prognosis. Finally, we investigated the association of the intendend treatment regimen (weekly versus 3-weekly; independent variable) with PFS and OS adjusted for the same confounders as listed above. This analysis was based on the total cohort of patients. In all multivariable models, patients with missing data were excluded, thus we only conducted completed case analyses. In all analyses a two-sided *p* < 0.05 was considered statistically significant. Statistical analysis was performed using the statistical program R (https://cran.r-project.org/).

## Results

### Patient characteristics

We included 314 eligible patients with a median age of 60 years. Patients’ clinical and demographic characteristics are summarized in Table 1. 127 (40.4%) patients were treated with cisplatin 100 mg/m^2^ in a 3-weekly schedule and 187 (59.6%) patients received cisplatin 40–50 mg/m^2^ weekly. The treatment groups were equally distributed with regard to clinical characteristics such as age, gender, tumor localisation, smoking history or comorbidities, with the exception of a slightly higher rate of patients with advanced stage (≥T2 and ≥ N2) SCCHN in the 3-weekly cisplatin group compared to the weekly cohort (64.2% vs. 51.1%). This numerical difference did not reach statistical significance.

**Table 1:** Patient characteristics

Baseline characteristic	3-weekly (*n* = 127)	Weekly (*n* = 187)	Total (*n* = 314)	*p*-value
Centre				< 0,0011
- USB	124 (97,6%)	0 (0%)	124 (39,5%)	
- KSW	3 (2,4%)	58 (32%)	61 (19,4%)	
- USZ	0 (0%)	129 (69%)	129 (41,1%)	
Age at diagnosis, mean (SD)	60.4 (8.1)	59.9 (8.7)	60.1 (8.5)	0.5582
Gender				0.2731
- Male	102 (80.3%)	139 (74.3%)	241 (76.8%)	
- Female	25 (19.7%)	48 (25.7%)	73 (23.2%)	
Tumor localisation				0.1561
- Oropharynx	57 (45.2%)	102 (54.8%)	159 (51%)	
- Oral cavity	30 (23.8%)	27 (14.5%)	57 (18.3%)	
- Hypopharynx	21 (16.7%)	27 (14.5%)	48 (15.4%)	
- Larynx	10 (7.9%)	21 (11.3%)	31 (9.9%)	
- CUP	5 (4.0%)	8 (4.3%)	13 (4.2%)	
- Nasopharynx	3 (2.4%)	1 (0.5%)	4 (1.3%)	
Smoking History				0.0871
- Smoker	105 (92.9%)	155(85.6%)	260 (88.4%)	
- Non smoker	8 (7.1%)	26 (14.4%)	34 (11.6%)	
HPV Status				0.0931
-Positive	21 (16.5%)	34 (18.2%)	55 (17.5%)	
- Negative	32 (25.2%)	29 (15.5%)	61 (19.4%)	
- Unknown	74 (58.3%)	124 (66.3%)	198 (63.1%)	
T Stage				0.0811
- T0	0	1 (0.6%)	1 (0.3%)	
- T1	14 (11.7%)	29 (16.2%)	43 (14.4%)	
- T2	36 (30.0%)	74 (41.3%)	110 (36.8%)	
- T3	31 (25.8%)	35 (19.6%)	66 (22.1%)	
- T4	39 (32.5%)	40 (22.3%)	79 (26.4%)	
N Stage				0.6271
- N0	24 (19.0%)	43 (23.0%)	67 (21.4%)	
- N1	13 (10.3%)	25 (13.4%)	38 (12.1%)	
- N2	83 (65.9%)	112 (59.9%)	195 (62.3%)	
- N3	6 (4.8%)	7 (3.7%)	13 (4.2%)	
M Stage				0,4823
- M0	115 (90.5%)	160 (85.5%)	275 (87.5%)	
- M1	5 (3.9%)	3 (1.6%)	8 (2.5%)	
Advanced Disease (> = T2 & > = N2)				0,0341
- Yes	77 (64,2%)	99 (51.1%)	169 (56.3%)	
- No	43 (35,8%)	88 (48,9%)	131 (43,7%)	
Type of CRT				0.1471
- Definitive	78 (61.4%)	126 (67.3%)	204 (64.9%)	
- Adjuvant	49 (38.6%)	61 (32.7%)	110 (35.1%)	

*SD* Standard deviation, ^1^Chi-Square test; ^2^t-test; ^3^Fisher’s exact test

### Treatment characteristics

127 patients were treated with a 3-weekly regimen. These were all patients treated at the USB (*n* = 124) and 3 patients treated at KSW. 187 patients received a weekly dose of cisplatin. At the USZ, 129 patients were treated with a weekly schedule (125 patients with 40 mg/m^2^ weekly and 4 patients with 30 mg/m^2^). Additionally, 17 patients received an induction chemotherapy before the cisplatin-based RCT, predominantly with taxanes, 5-fluorouacil and platinum. At the KSW 58 patients were treated with a weekly schedule (50 mg/m^2^ in 58 patients and 40 mg/m^2^ in one patient) and 3 patients received a 3-weekly schedule.

### Treatment regimen and cumulative cisplatin dose

**Fig. 1:**
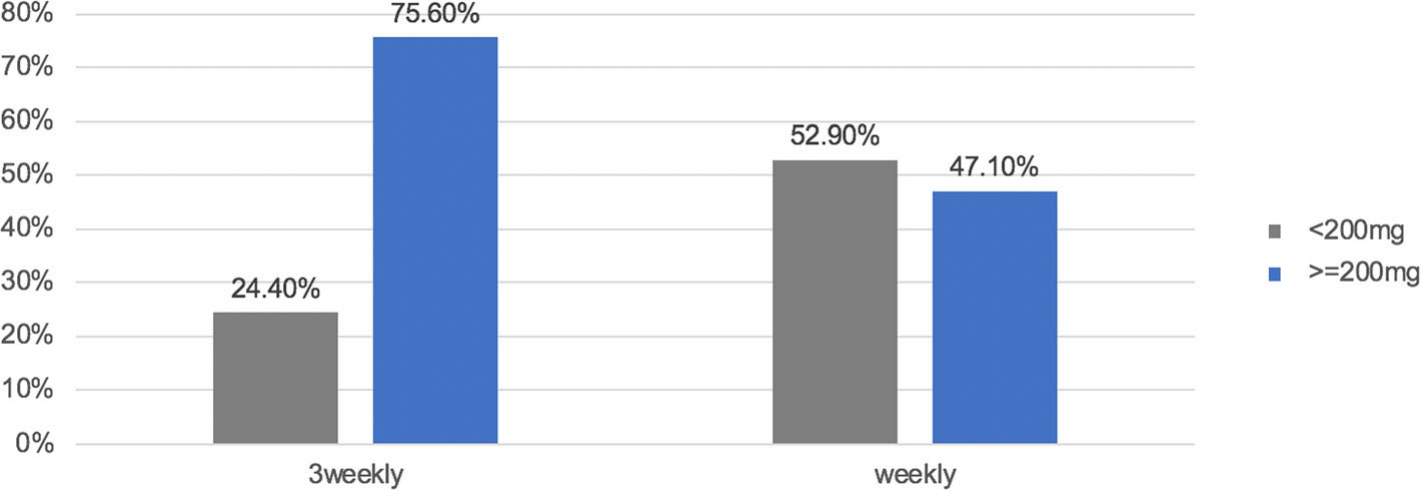
Cumulative cisplatin dose with a cut-off dose of 200 mg/m^2^ comparing 3-weekly vs. weekly schedule

More patients were able to receive a higher cumulative dose of at least 200 mg/m^2^ if given at a 3-weekly dose compared with those receiving weekly cisplatin (*N* = 96 (75.6%) vs. *N* = 88 (47.1%, *p* < 0.001) (Fig. 1 and Table 2). Median cumulative cisplatin dose was 200 mg/m^2^ (IQR 150–300) for patients treated with a 3-weekly schedule and 160 mg/m^2^ (120–240) for the weekly schedule (*p* < 0.001). With regard to chemotherapy compliance, 47 patients (37%) of patients treated with a 3-weekly regimen completed the full 3 cycles of treatment and 46 patients (36%) received 2 cycles. 33 patients (25.9%) were changed to a different chemotherapy regimen, most of them to carboplatin (31 patients, 24.4%). Of patients who received weekly chemotherapy the number of patients who managed to complete six, five, four and three cycles was 22 (11.7%), 42 (22.4%), 65 (34.7%) and 19 (10.1%), respectively. In the weekly treatment cohort, 37 patients (18.7%) had to change to a different chemotherapy due to ineligibility for cisplatin in the course of the treatment. 32 patients (17.1%) switched to cetuximab and 3 patients (1.6%) to carboplatin. Dose reductions due to adverse events were necessary in 6/127 patients (4.7%) receiving cisplatin 3-weekly and 11/187 patients (5.8%) with a weekly schedule.

**Table 2:** Cisplatin Dose Intensity

	3-weekly Cisplatin	Weekly Cisplatin	*p*-value
Median cumulative cisplatin dose	200 mg/m^2^	160 mg/m^2^	< 0.001
Cumulative dose ≥200 mg^2^	96 (75.6%)	88 (47.1%)	< 0.001
Dose reduction	6 (4.7%)	11 (5.8%)	
Change to other chemotherapy	33 (25.9%)	35 (18.7%)	

In the multivariable logistic regression analysis, patients receiving the the 3-weekly treatment regimen were much more likely to reach the cumulative cisplatin dose of ≥200 mg/m^2^ (odds ratio (OR) 3.46, 95%CI 2.10–5.69), irrespective of age and gender (Table 3).

**Table 3:** Multivariable logisitic regression analysis for Cumulative Cisplatin Dose

Factor	Odds ratio	95% Confidence interval	*p*-value
Treatment regimen	3.46	2.10–5.69	< 0.0001
Gender	1.22	0.70–2.13	0.465
Age	0.99	0.96–1.02	0.696

### Outcome, progression free survival and overall survival

**Fig. 2:**
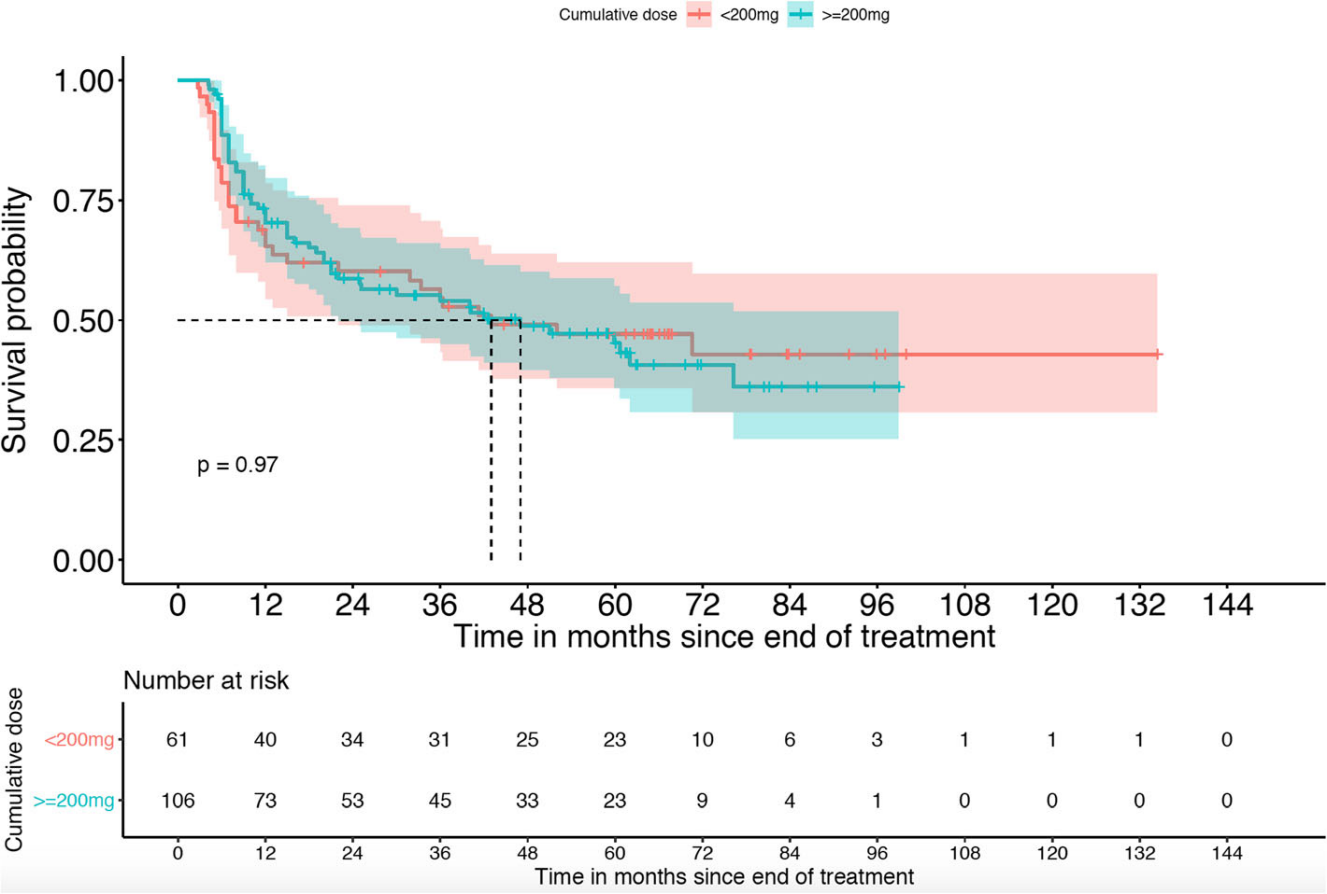
Comparison of treatment regimens (3-weely vs. weekly cisplatin) for progression-free survival (landmark analysis)

**Fig. 3:**
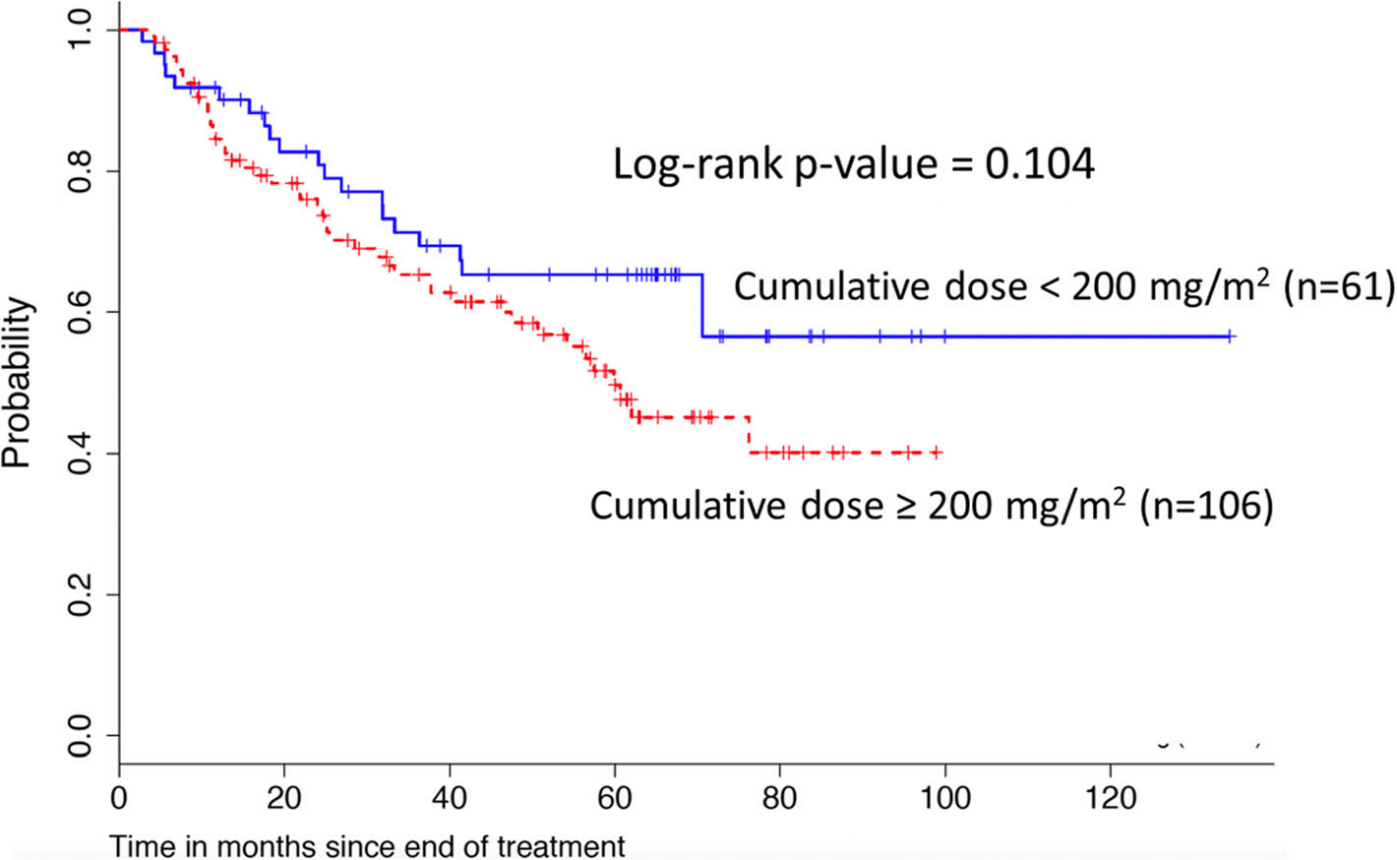
Comparison of treatment regimens (3-weely vs. weekly cisplatin) for overall survival (landmark analysis)

Median follow-up time was 40.6 months (range, 1–134 months). At the time of analysis, 120 patients (61.8%) had a disease progression or a relapse and 198 patients were still alive. Median OS for the whole cohort was 83.8 months (95%CI 76.6–91.1). In our landmark analysis, we could not confirm that a cisplatin dose ≥200 mg/m^2^ is associated with better PFS (HR 0.9, 95% CI 0.7–1.3) (Fig. 2, Table 4) or OS (HR 1.3, 95% CI 0.8–1.9) (Fig. 3, Table 5). There was some evidence that current/former smokers and older patients had a poorer outcome (Table 4); a similar pattern was seen for OS (Table 5). Considering the whole cohort, there was no evidence of a difference between the intended treatment regimens (weekly versus 3-weekly) and PFS (Table 6) or OS (Table 7).

**Table 4:** Multivariable Cox regression analysis for PFS including cumulative cisplatin dose; landmark analysis only including patients alive 8 weeks after end of treatment

Factor	Hazard Ratio	95% Confidence interval	p-value
Cumulative cisplatin dose	0.92	0.65–1.29	0.641
Gender	1.10	0.74–1.65	0.617
Age at diagnosis	1.01	0.99–1.03	0.089
Hypopharyngeal localisation	0.88	0.56–1.37	0.582
Smoking history	2.25	1.13–4.47	0.02

**Table 5:** Multivariable Cox regression analysis for OS including cumulative cisplatin dose; landmark analysis only including patients alive 8 weeks after end of treatment

Factor	Hazard ratio	95% Confidence interval	*p*-value
Cumulative cisplatin dose	1.25	0.84–1.87	0.263
Gender	1.05	0.66–1.67	0.821
Age at diagnosis	1.02	1.00–1.05	0.012
Hypopharyngeal localisation	1.09	0.66–1.80	0.735
Smoking history	1.98	0.91–4.31	0.082

**Table 6:** Multivariable Cox regression analysis for PFS; whole cohort

Factor	Hazard ratio	95% Confidence interval	*p*-value
Treatment regimen (weekly vs. 3-weekly)	0.89	0.62–1.26	0.515
Gender	1.11	0.74–1.66	0.744
Age at diagnosis	1.01	0.99–1.03	0.071
Hypopharyngeal localisation	0.88	0.57–1.38	0.603
Smoking history	2.29	1.15–4.53	0.017

**Table 7:** Multivariable Cox regression analysis for OS; whole cohort

Factor	Hazard Ratio	95% Confidence interval	*p*-value
Treatment regimen (weekly vs. 3-weekly)	1.37	0.92–2.03	0.116
Gender	1.08	0.68–1.71	0.741
Age at diagnosis	1.02	1.00–1.05	0.017
Hypopharyngeal localisation	1.03	0.62–1.70	0.893
Smoking history	2.00	0.92–4.33	0.078

### Adverse events and toxicity

The comparison of both cisplatin regimens revealed that the 3-weekly schedule of cisplatin was associated with significantly higher rate of renal toxicity (33.1% vs. 20.9%, *p* = 0.016) (Table 4). The rate of ototoxicity was similar in both groups (15% vs. 12.8%, *p* = 0.60). Assisted feeding in the form of a feeding tube was used in 81 patients (63.7%) in the 3-weekly regime and 105 patients (56.1%) in the weekly regimen. In the multivariable regression model, patients receiving the 3-weekly treatment regimen were at higher risk for nephrotoxicity (OR 1.88, 95% CI 1.12–3.16, p = 0.016) and this also increased with age (OR 1.03, 95%CI 1.00–1.07, *p* = 0.028). None of these factors were associated with ototoxicity (Table 8).

**Table 8:** Cisplatin treatment regimen and acute toxicity

Toxicity	3-weekly (*n* = 127)	Weekly (*n* = 187)	Total (*n* = 314)	*p*-value
Nephrotoxicity				0.016
Yes	42 (33.1%)	39 (20.9%)	81 (25.8%)	
No	85 (66.9%)	148 (79.1%)	233 (74.2%)	
Ototoxicity				0.711
Yes	19 (15%)	24 (12.8%)	43 (13.7%)	
No	108 (85%)	163 (87.2%)	271 (86.3%)	

## Discussion

This is one of the largest retrospective studies evaluating different treatment regimens for combined radio-chemotherapy in SCCHN patients undergoing combined RCT with cisplatin. We analysed SCCHN patients treated at three different sites in Switzerland. One advantage of this study was, that treatment allocation was done by site (based on internal guidelines) and not on patient-based criteria, thus excluding an important selection bias. Patients treated according to the 3 weekly cisplatin schedule were more likely to achieve a cumulative cisplatin dose of ≥200 mg/m^2^. However, neither the treatment regimen (three weekly versus once weekly) nor the cumulative cisplatin dose of ≥200 mg/m^2^ was associated with improved PFS or OS. However, the weekly regimen was associated with less renal toxicity.

Most randomized trials investigating the role of concurrent cisplatin-based RCT used a three weekly schedule of cisplatin 100 mg/m^2^ and this treatment regimen is considered the standard therapy in LA-SCCHN patients. However, it is associated with substantial toxicity and many trials showed suboptimal compliance with cisplatin 100 mg/m^2^ potentially negatively influencing the outcome [5, 17, 37]. Therefore, low-dose weekly cisplatin schedules are frequently used in clinical practice despite the lack of evidence from prospective randomized trials [19, 21, 24, 26–28, 38, 39].

In a pooled analysis from two tertiary academic cancer centres 659 patients with stage III/IV SCCHN treated with single-agent cisplatin RCT were analysed and a survival benefit for patients receiving a cisplatin dose > 200 mg/m^2^ was shown in HPV negative LA-SCCHN [40]. Therefore, a cumulative cisplatin dose of > 200 mg/m^2^ is usually pursued in this setting.

In our study, treatment compliance was lower with the weekly regimen as reflected by a significantly lower cumulative cisplatin dose. With a weekly regimen less than half of patients achieved a cumulative dose of > 200 mg/m^2^ whereas this was the case for three-fourths of patients treated with a 3-weekly regimen. We hypothesize that the flexibility to decide on treatment continuation on a weekly basis might lead to more frequent treatment discontinuation even with low-grade toxicities whereas with a three weekly schedule this decision can only be taken at two time points and many patients might have recovered from chemotherapy associated side effects after three weeks. The retrospective analysis by Geiger et al. was limited to stage III/IV SCCHN patients who had surgery and were treated with adjuvant RCT and compared a conventional three weekly regimen (100 mg/m^2^) to weekly cisplatin with a dose of 25–30 mg/m^2^ [31]. Of 104 patients, 51 received a three weekly regimen. Similar to our study, the median cisplatin dose was higher with the three weekly regimen. In the retrospective comparison by Rades et al. the proportion of patients achieving a cumulative cisplatin dose of > 200 mg/m^2^ was not different for the two RCT groups [27]. This study was limited to patients undergoing definitive RCT and did not allow for adjuvant RCT therefore representing a more homogenous patient population. Overall, compared to our study much fewer patients reached the threshold of 200 mg/m^2^ with 32 and 41% of patients in the weekly and three weekly group, respectively. The lower cumulative cisplatin dose in the weekly cisplatin group may partially be explained by the weekly dose of 30 mg/m^2^ used in a part of patients. The authors did not provide exact numbers for patients receiving 30 mg/m^2^ or 40 mg/m^2^ weekly, respectively. In our study, two third of patients were treated with a weekly dose of 40 mg/m^2^ and 30% of patients even received a weekly dose of 50 mg/m^2^. Also the randomized trial by Noronha used a weekly cisplatin dose of 30 mg/m^2^ [23]. This dose is frequently used in clinical practice based on retrospective data [38, 41, 42] and two randomized trials from the Indian group [43, 44]. Our study provides additional evidence that the 200 mg/m^2^ cumulative dose can be reached more often by using a three weekly regimen of cisplatin. The more important question of course is, whether this threshold is a valid surrogate marker for PFS or OS.

We did not find an impact of the treatment regimen on the outcome. A recent systematic review compared 4 prospective studies with weekly cisplatin regimen to 7 prospective studies using a 3-weekly high-dose cisplatin treatment and reported superior outcome for the high-dose cisplatin treatment [18]. A small randomized study compared 100 mg/m^2^ cisplatin every 3 weeks to weekly 40 mg/m^2^ [34]. This trial was limited to patients with oral cavity tumors and did not show differences in locoregional control and survival. In addition, with only 50 patients enrolled, this small trial was underpowered to show potential differences. Recently, a large randomized non-inferiority trial (*N* = 300) compared cisplatin 30 mg/m^2^ given once a week and cisplatin 100 mg/m^2^ given once every 3 weeks concurrently with curative intent RT was published [23]. The primary aim to show non-inferiority for the weekly regimen was not reached. In fact, locoregional tumor control was superior with the high-dose 3-weekly regimen. Most patients (93%) included in the trial had tumor resection before and RCT was given as adjuvant therapy. Moreover, the trial population consisted mainly of oral cavity tumors (87.3%) in patients with smokeless tobacco consumption (71.3%). Only 19.7% of patients were smokers. In our study, oral cavity cancers only account for 18.3% of all tumors and 88.4% of patients were smokers. It is therefore questionable if the results of the randomized trial from a large academic hospital in India are applicable for Western patient populations with a predominance of smoking-associated pharyngeal carcinomas.

Retrospective studies comparing weekly and three weekly RCT regimens demonstrated conflicting results. The trial by Espeli et al. with 94 patients demonstrated improved OS and similar PFS with cisplatin 100 mg/m^2^ every 3 week compared to 40 mg/m^2^ weekly [30]. Importantly, patients in the weekly cisplatin group were significantly older introducing a potential bias for outcome and toxicities. As in our study cumulative cisplatin dose was significantly lower with the weekly regimen. Significantly more patients in the high-dose cisplatin group developed chronic renal failure whereas in our trial differences in renal toxicity was limited to the acute phase. The retrospective comparison by Rades et al. also showed better locoregional control and OS when using cisplatin 100 mg/m^2^ every three weeks compared to 30–40 mg/m^2^ weekly in 133 patients with LA-SCCHN [27]. In the previously discussed study by Geiger et al. there was no difference in loco-regional control but a trend towards a better survival with 3-weekly cisplatin [31].

In our study, 65% of patients were treated with curative intent definitive RCT, the others received RCT in the adjuvant setting. The heterogeneity of treatment regimens may have confounded our results although the distribution between definitive and adjuvant RCT was similar in the high- and low-dose cisplatin groups in our study. Another limitation of our study is that HPV status was only available in 116 patients (36.9%). Proportions of HPV positive patients were similar in both RCT groups but heterogeneity based on missing HPV analysis in the majority of patients cannot be excluded. The study by Spreafico et al. showed an impact of a cumulative cisplatin dose > 200 mg/m^2^ in patients with HPV negative LA-SCCHN therefore suggesting different sensibility to cisplatin in general or the dose intensity of cisplatin. In the previously discussed analysis by Geiger et al. 50% of patients had HPV-associated oropharyngeal cancer [31]. Although there was a trend towards a better survival for patients treated with a 3-weekly regimen, this difference was not seen in the subgroup of HPV positive patients suggesting that a less intense treatment regimen might be sufficient for patients with HPV-associated LA-SCCHN.

In our study, we found significantly higher renal toxicity with a 3-weekly cisplatin regimen. Interestingly, results from previously discussed studies report conflicting results with regard to toxicity. Several studies reported higher toxicity for 3-weekly cisplatin, mainly renal toxicity [26–28, 30], but also hematotoxicity [27] and mucositis/dermatitis [28]. In contrast, Tsan et al. reported a higher rate of mucositis and overall toxicity in the weekly cisplatin group [34]. Also the recent comparative analysis of different prospective trials showed less toxicities with the 3-weekly regimen [18]. The fact that this is a retrospective study limits the value of toxicity assessment as we were not able to perform a comprehensive adverse event and toxicity assessment. We therefore decided to investigate oto- and nephrotoxicity as two of the main long-term toxicities in patients treated with cisplatin.

Existing results comparing three weekly and weekly cisplatin regimen combined with RT are conflicting and because they are retrospective they always bear the risk of selection bias. The only statistically powered prospective randomized trial did not prove non-inferiority for a weekly regimen [23]. However, based on the previously discussed issues the validity of the trial for LA-SCCHN in Western countries is questionable. Our study shows a significant higher cumulative cisplatin dose with a 3-weekly regimen. However, we couldn’t find differences in survival outcomes challenging recent data suggesting the prognostic impact of a cumulative cisplatin dose higher than 200 mg/m^2^ [40]. The retrospective design of this large study with all its inherent problems and potential biases makes it difficult to draw definitive conclusions from the analysis. We made every attempt to assess the biases by using multivariable analyses. However, further prospectively randomized trials are needed to establish the most effective RCT treatment regimen. Currently, a randomized phase 3 trial comparing cisplatin 40 mg/m^2^ weekly with 100 mg/m^2^ every three weeks combined with RT is ongoing [45].

## Conclusions

This retrospective, multicentre study with more than 300 patients demonstrates that significantly more patients receive a cumulative of dose of ≥200 mg/m^2^, when treated with a 3-weekly schedule compared to weekly dosing. However, we could not find differences in survival between the regimens. Furthermore, we could not confirm previous data suggesting that a cumulative cisplatin dose ≥200 mg/m^2^ is associated with better survival. The 3-weekly regimen led to a higher rate of renal toxicity. Although a 3-weekly regimen allows for a higher cumulative cisplatin dose, weekly cisplatin may be an acceptable alternative treatment considering toxicity. Well-designed prospective trials are needed to establish the most effective RCT treatment regimen and to define patient subgroups that need more intense treatment regimens.

## Abbreviations

AE: Adverse event
CI: Confidence interval
CT: Computertomography
HPV: Human papilloma virus
HR: Hazard ratio
IMRT: Intensity modulated radiotherapy
IQR: Interquartile range
KSW: Kantonsspital Winterthur (Cantonal Hospital Winterthur)
LA-SCCHN: Locally advanced squamous cell carcinoma of the head and neck
OR: Odds ratio
OS: Overall survival
PET: Positron emission tomography
PFS: Progression-free survival
Pts: Patients
RCT: Radio-chemotherapy
RT: Radiotherapy
SCCHN: Squamous cell carcinoma of the head and neck
USB: University Hospital Basel
USZ: University Hospital Zürich
